# Dengue Fever Surveillance in Mato Grosso do Sul: Insights from Genomic Analysis and Implications for Public Health Strategies

**DOI:** 10.3390/v15091790

**Published:** 2023-08-23

**Authors:** Larissa Domingues Castilho de Arruda, Marta Giovanetti, Vagner Fonseca, Marina Castilhos Souza Umaki Zardin, Gislene Garcia de Castro Lichs, Silvia Asato, Ana Olivia Pascoto Esposito, Miriam Tokeshi Müller, Joilson Xavier, Hegger Fritsch, Mauricio Lima, Carla de Oliveira, Elaine Vieira Santos, Livia de Mello Almeida Maziero, Danila Fernanda Rodrigues Frias, Danielle Ahad das Neves, Liliane Ferreira da Silva, Ellen Caroline Rodrigues Barretos, Paulo Eduardo Tsuha Oshiro, Bianca Modafari Goday, Jéssica Klener Lemos dos Santos, Simone Kashima, Carlos F. C. de Albuquerque, Rodrigo Fabiano do Carmo Said, Alexander Rosewell, Luiz Henrique Ferraz Demarchi, Julio Croda, Luiz Carlos Junior Alcantara, Crhistinne Cavalheiro Maymone Gonçalves

**Affiliations:** 1 Secretaria de Estado de Saúde de Mato Grosso do Sul, Campo Grande 79031-350, MS, Brazil; larissacastilhodocs@gmail.com (L.D.C.d.A.); mellolivia12@hotmail.com (L.d.M.A.M.); danila.frias@saude.ms.gov.br (D.F.R.F.); danielle.neves@saude.ms.gov.br (D.A.d.N.); enf.lilianesilva@gmail.com (L.F.d.S.); ellencarol1234567@gmail.com (E.C.R.B.); pauloedu.oshiro@gmail.com (P.E.T.O.); bianca.modafari@hotmail.com (B.M.G.); doencasendemicasms@outlook.com (J.K.L.d.S.); 2 Universidade Federal do Estado do Mato Grosso do Sul, Campo Grande 79070-900, MS, Brazil; 3 Instituto Rene Rachou, Fundação Oswaldo Cruz, Belo Horizonte 30190-002, MG, Brazil; joilsonxavier@live.com (J.X.); hegger.fritsch@gmail.com (H.F.); maurili15@hotmail.com (M.L.); 4 Sciences and Technologies for Sustainable Development and One Health, Università Campus Bio-Medico di, 00128 Roma, Italy; 5 Coordenação de Vigilância, Preparação e Resposta à Emergências e Desastres (PHE), Organização Pan-Americana da Saúde/Organização Mundial da Saúde (OPAS/OMS), Brasília 70312-970, DF, Brazil; vagnerfonseca@gmail.com; 6 SES-MS-Laboratório Central de Saúde Pública de Mato Grosso do Sul, Campo Grande 79031-350, MS, Brazil; ninaumaki@gmail.com (M.C.S.U.Z.); glichs@hotmail.com (G.G.d.C.L.); silvia.asato@saude.ms.gov.br (S.A.); miriam.muller@saude.ms.gov.br (M.T.M.); lhdemarchi@uol.com.br (L.H.F.D.); 7 lnstituto Oswaldo Cruz, Fundação Oswaldo Cruz, Rio de Janeiro 21040-900, RJ, Brazil; oliveirasc@yahoo.com.br; 8 Fundação Hemocentro de Ribeirão Preto, Ribeirão Preto 14051-140, SP, Brazilskashima@hemocentro.fmrp.usp.br (S.K.); 9 Organização Pan-Americana da Saúde, Organização Mundial da Saúde, Brasília 70312-970, DF, Brazil; carloiscampelo@gmail.com (C.F.C.d.A.); saidrod@paho.org (R.F.d.C.S.); rosewelale@paho.org (A.R.); 10 Fundação Oswaldo Cruz, Universidade Federal de Mato Grosso do Sul-UFMS, Campo Grande 79000-000, MS, Brazil; juliocroda@gmail.com

**Keywords:** dengue virus, genomic surveillance, phylodynamics, epidemiology

## Abstract

Since its discovery in early 1916, dengue fever, a common vector-borne illness in Brazil, has resulted in extensive urban outbreaks and poses a serious threat to the public’s health. Understanding the dynamics of Dengue Virus (DENV) serotypes circulating in different regions of Brazil is essential for implementing effective disease control and prevention measures. In response to this urgent need, we conducted an on-site training program in genomic surveillance in collaboration with the Central Laboratory of Health and the Secretary of Health of the Mato Grosso do Sul state. This initiative resulted in the generation of 177 DENV genome sequences collected between May 2021 and May 2022, a period during which over 11,391 dengue fever cases were reported in the state. Through this approach, we were able to identify the co-circulation of two different dengue serotypes (DENV1 and DENV2) as well as the existence of diverse viral lineages within each genotype, suggesting that multiple introduction events of different viral strains occurred in the region. By integrating epidemiological data, our findings unveiled temporal fluctuations in the relative abundance of different serotypes throughout various epidemic seasons, highlighting the complex and changing dynamics of DENV transmission throughout time. These findings demonstrate the value of ongoing surveillance activities in tracking viral transmission patterns, monitoring viral evolution, and informing public health actions.

## 1. Introduction

Dengue virus (DENV), a mosquito-borne virus from the Flaviviridae family, is classified into four antigenically distinct and genetically related serotypes (DENV1-4) [1]. Each serotype comprises multiple phylogenetically defined genotypes, with nucleotide divergence typically below 6% [1,2]. These genotypes often exhibit different spatiotemporal distributions. Surveillance of circulating serotype diversity is crucial for public health, as heterotypic infections within the same serotype pose the greatest risk for severe clinical outcomes [1,2]. DENV is endemic to many tropical and subtropical regions worldwide, posing a significant public health threat [1,2]. Approximately 50% of the global population (nearly 4 billion people) is at risk of annual infections [2]. Diagnosing dengue can be challenging due to clinical similarities with other febrile illnesses [3,4,5]. Clinical manifestations of dengue range from asymptomatic or mild infection to more severe forms such as dengue fever, hemorrhagic fever, and dengue shock syndrome [6]. Laboratory tests for diagnosis include direct methods (viral isolation by culture, detection of nucleic acids by reverse transcriptase PCR [RT-PCR], and identification of the dengue nonstructural glycoprotein 1 [NS1] antigen secreted by infected cells) and indirect methods (detection of IgM and IgG antibodies by ELISA) [7,8]. 

Designated as an acute febrile infectious disease of viral origin by the World Health Organization (WHO), dengue is the most common arbovirus affecting people and a serious public health concern, particularly in the Americas [9]. Dengue fever incidence has increased dramatically in the Americas during the last four decades [9,10]. Starting from 1.5 million cases during the 1980s, the numbers have surged dramatically to an alarming 16.2 million cases between 2010 and 2023 [9,10]. This escalating trend has been accompanied by significant Dengue outbreaks within the WHO Region of the Americas, with the year 2023 marking a notable milestone [9,10]. Within this year alone, the number of suspected and confirmed Dengue cases has surpassed an alarming three million, exceeding the total of 2.8 million cases reported throughout the entirety of 2022 [10]. 

Notably, despite growing worried about other mosquito-borne infections such as Zika and chikungunya, DENV continues to have a significant impact in the region [11,12,13,14]. In 2023, the highest number of dengue cases was observed in Brazil, accounting with a total number of 2,376,522 cases, followed by Peru and Bolivia [13,14]. Despite the various measures put in place to control the spread of arboviral diseases such as the dengue virus, there is still a significant knowledge gap regarding the prevalence and circulation of different dengue strains in several regions across Brazil, including the Midwest. This region has a unique network of connectivity with other countries and regions of Brazil, increasing the possibility of disease transmission across borders. This is especially significant considering the constant flow of people, trade, and travel via these interconnected regions. Furthermore, the Pantanal biome within the midwestern region adds an additional level of complexity. With its unique ecosystem, the Pantanal might potentially act as a reservoir for disease vectors, offering a perfect habitat for dengue transmission. Furthermore, the midwestern region appears ideal for the disease’s transmission cycle due to its climate which might likely promotes both vector multiplication and viral replication. In order to fill this knowledge gap, we established an on-site genomic surveillance training program in partnership with the Central Laboratory of Health and the Secretary of Health of Mato Grosso do Sul state. Through this initiative, we successfully generated 177 DENV genome sequences collected between May 2021 and May 2022. Notably, this period witnessed the reporting of over 11,391 dengue fever cases in the state. These sequences have played a pivotal role in comprehensively monitoring the circulation of different DENV serotypes and genotypes in the region. Our analysis has unveiled a complex dynamic history characterized by multiple introduction events, and co-circulation of different viral strains throughout different epidemic seasons. These findings further underscore the critical importance of genomic surveillance in tracking the real-time evolution of circulating viral forms and facilitating early detection of potential novel strains. 

## 2. Materials and Methods

### 2.1. Ethics Statement

The Oswaldo Cruz Foundation Ethics Committee (CAAE: 90249218.6.1001.5248) and the Pan American Health Organization Ethics Evaluation Committee (Ref. No. PAHO-2016-08-0029) both approved this study effort after comprehensive evaluation. The availability of these samples for research purposes during outbreaks of national concern is permitted under the terms of the 510/2016 Resolution of the National Ethical Committee for Research–Brazilian Ministry of Health (CONEP—Comisso Nacional de Ética em Pesquisa, Ministério da Sade), which authorizes the use of clinical samples collected in the Brazilian Central Public Health Laboratories to accelerate knowledge building without the requirement of informed consent. The samples used in this investigation were collected anonymously from material that went beyond regular arbovirus identification in Brazilian public health laboratories that are part of the public network within BrMoH.

### 2.2. Collection of Samples and Molecular Testing

A total of 177 residual serum clinical samples were collected from patients who sought medical care spontaneously during the epidemic season. These patients exhibited symptoms consistent with DENV infection. These samples come with accompanying epidemiological metadata, including details like the date of symptom onset, date of sample collection, gender, age, municipality of residence, exhibited symptoms, and disease classification. The collection was conducted by the Secretary of Health from May 2021 to May 2022. Subsequently, these samples were sent to the Public Central Health Laboratory of the state of Mato Grosso do Sul for molecular screening. To isolate the viral RNA, the QIAmp Viral RNA Mini Kit from Qiagen was employed for all clinical samples. Detection of DENV1-4 was performed using reverse transcription quantitative polymerase chain reaction (RT-qPCR) analysis [15].

### 2.3. Genome Sequencing and cDNA synthesis

All DENV1 (*n* = 172) and DENV2 (*n* = 5) positive samples were sent for genome sequencing based on the RT-qPCR cycle threshold (Ct). The SuperScript IV Reverse Transcriptase kit (Thermo Fisher Scientific, Waltham, MA, USA) was used for the synthesis of the cDNA, which was then submitted to multiplex PCR (35 cycles) using Q5 High Fidelity Hot-Start DNA Polymerase (NEB) and a set of specific primers as previously mentioned [1]. Amplicons were purified with 1× AMPure XP Beads (Beckman Coulter, Brea, CA, USA) and measured using QubitTM dsDNA HS Assay Kit (Thermofisher Scientific) on a Qubit 3.0 fluorimeter. Following the previously reported reaction conditions [1], DNA library preparation was carried out using the Oxford Nanopore Technologies Ligation Sequencing kit and Native Barcoding Expansion 1-96 kit [15]. Sequencing was carried out using a MinION device for up to 24 h. We employed negative controls in each sequencing run to prevent and check for any contamination with less than 2% mean coverage.

### 2.4. Generation of Consensus Sequences

Guppy v3.4.5 was used to basecall raw data, while qcat was used to demultiplex barcodes. Consensus sequences were generated by de novo assembling using Genome Detective (https://www.genomedetective.com/, accessed on 27 July 2023) [16]. Using the viral portion of the Swissprot UniRef [16] protein database, Genome Detective uses DIAMOND to discover and classify possible viral readings in broad taxonomic groupings. Following that, using NCBI blastn, candidate reads are assigned to candidate reference sequences and aligned using AGA (Annotated Genome Aligner) and MAFFT. Finally, the final contigs and consensus sequence are available as a FASTA file. More information on Genome Detective can be found in [16]. The new sequences reported in this study (OR258403–OR258579) were igenotyped using the arbovirus phylogenetic subtyping tool, which is available at http://genomedetective.com/app/typingtool/dengue; this confirmed that the newly genotypes belonged to DENV1-V and DENV2-III, respectively.

### 2.5. Phylogenetic Reconstruction

The DENV1 (*n* = 172) and DENV2 (*n* = 5) complete genome sequences generated in this study were combined with globally sampled and publicly available DENV1 genotype V (DENV1-V = 552) and DENV2 genotype III (DENV2-III = 648) complete genome sequences that were retrieved from NCBI up to May 2022 to study the evolution of DENV1-2 in the state of Mato Grosso do Sul. Sequences that accounted for less than 50% of the viral genome as well as those without a sample date or location were discarded. Multiple sequence alignment was performed using MAFFT [17] and manually curated to remove artefacts using Aliview [18]. The GTR nucleotide substitution model, which was determined to be the best-fit model by the Model Finder application built into IQ-TREE2, was used to estimate the maximum Likelihood (ML) phylogenetic trees [19]. The tree topology’s robustness was assessed using 1000 bootstrap replications. The RDP4 tool was used to test for recombination signals prior to any phylogenetic analysis, but no evidence of recombination was found [20]. Using the BEAST (v.1.10.4) application, time-scaled phylogenetic trees were inferred on identified community transmission clades after the presence of a temporal signal that was assessed in TempEst [21,22]. Analyses were run in duplicate for 100 million MCMC steps, sampling parameters, and trees every 10,000th step Each run’s convergence was evaluated using Tracer (effective sample size for all pertinent model parameters >200). Using TreeAnnotator, the MCC trees for each run were compiled after the initial 10% was discarded as burn-in. To determine the best suitable molecular clock model for the Bayesian phylogenetic analysis, we used a strict model selection technique that included path-sampling (PS) and stepping-stone (SS) procedures [23]. The uncorrelated relaxed molecular clock model was chosen by estimating marginal likelihoods using the codon-based SRD06 model of nucleotide substitution and the nonparametric Bayesian Skyline coalescent model.

### 2.6. Epidemic Curves Based on Cases of Dengue Fever Recorded in Mato Grosso do Sul

Data on the serotype prevalence and weekly reported cases of dengue fever in the state of Mato Grosso do Sul from 2011 to 2022 were supplied by the Brazilian Ministry of Health of Brazil (BrMoH) (Ministério da Saúde, Brasilia, DF, Brazil 2022). These data were used to calculate incidence and to plot time series charts.

## 3. Results

**Figure 1:**
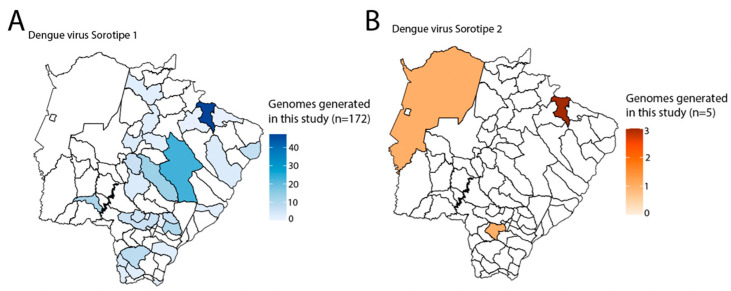
Genomics and epidemiological reconstruction of DENV1-2 in the state of Mato Grosso do Sul. (**A**,**B**) Spatial distribution of DENV1-2 genome sequences (*n* = 177 for DENV1 and *n* = 5 for DENV2) obtained in this study.

A total of 177 samples were screened using RT-qPCR, and they tested positive for either DENV1 (*n* = 172) or DENV2 (*n* = 5). All tested samples had sufficient DNA (≥2 ng/μL) for library preparation. The average PCR cycle threshold (Ct) values for DENV1 were 19 (range: 14 to 28), while for DENV2, they were 25 (range: 24 to 28). Table S1 provides additional epidemiological details of the processed samples. To obtain the complete genome sequences, we employed a portable nanopore sequencing method on the 177 viral samples. The sequencing process yielded an average coverage of 91% for DENV1 and 70% for DENV2, as detailed in Table S1 and were classified as DENV1 genotype V and DENV2 genotype III. The DENV1 sequences were obtained from a total of 30 different municipalities (Figure 1A) and covered the period from March to May 2022. On the other hand, the DENV2 samples were collected from three distinct municipalities (Figure 1B) between May and July 2021, as shown in Table S1. Regarding the age distribution, the median age of individuals infected with DENV1 was 36 years, ranging from 4 to 78 years. For DENV2, the median age was 30 years, ranging from 12 to 43 years. It is noteworthy that all the collected samples were obtained from patients who did not exhibit any alarming clinical signs or symptoms associated with the infection.

**Figure 2:**
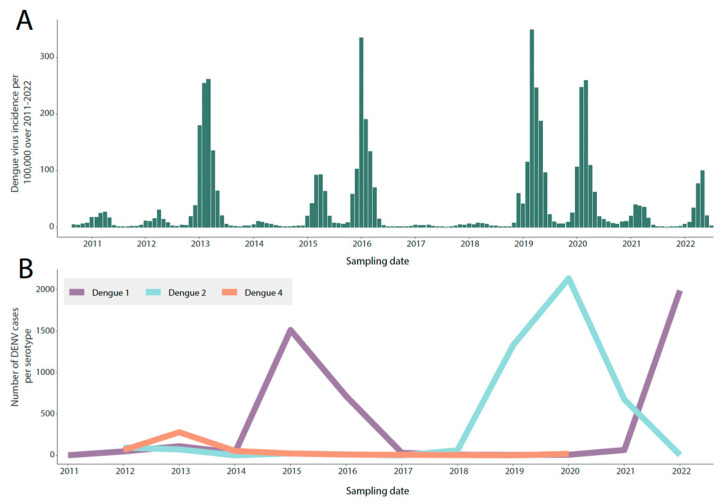
Annual Number of Dengue Virus (DENV) Cases by Serotype Reported in Mato Grosso do Sul State from 2011 to 2022. (**A**) Time series showing the monthly reported cases of dengue fever normalized per 100,000 individuals in the state of Mato Grosso do Sul from 2011 to 2022; (**B**) Proportion (percentage) of dengue cases by serotype reported in Mato Grosso do Sul State from 2011 to 2022.

Figure 2 illustrates the weekly cases of DENV normalized per 100,000 individuals reported in the state of Mato Grosso do Sul between 2011 and 2022. During this period, four major outbreaks of dengue fever occurred: in late 2013, 2016, 2019, and 2020. Additionally, a reduction in the number of cases was observed in 2017 and 2018. This decline can be attributed to the gradual accumulation of herd immunity (Figure 2). Starting from 2011, the dengue virus serotypes detected in the state were DENV1, DENV2, and DENV4. However, in 2013, DENV4 became the most common serotype, accounting for 60% of all cases (280 out of 465). Following that, there was a significant increase in DENV1 cases in the region, with a peak accounted in 2015 when a total of 1513 cases were registered. In early 2018, DENV2 replaced the previously circulating strain, resulting in over 3538 cases between 2018 and 2020. Subsequently, another displacement occurred, with DENV1 becoming dominant again in the region. This led to a new peak caused by the DENV1 genotype in 2022, which accounted for 1989 cases. 

### 3.1. DENV1 Phylodynamics in the State of Mato Grosso do Sul, Midwest Brazil

**Figure 3:**
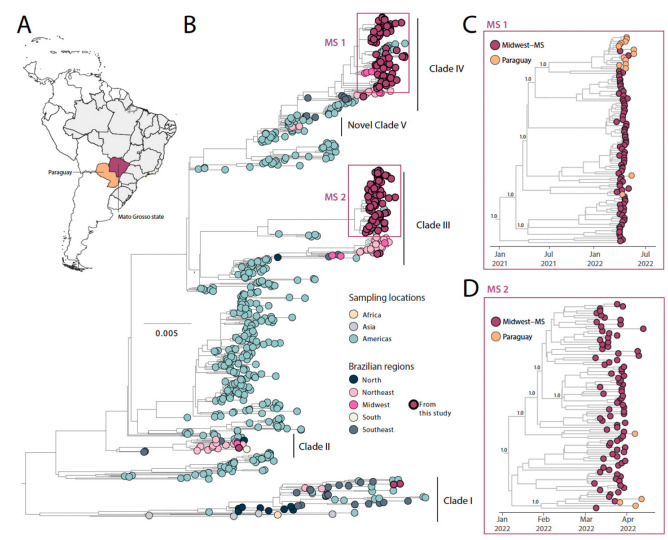
Genomic monitoring of DENV1 genotype V in Mato Grosso do Sul state, Midwest Brazil. (**A**) Map showing the spatial area under investigation; (**B**) Maximum likelihood (ML) phylogenetic analysis of 172 complete genome sequences from DENV1 generated in this study plus *n* = 552 available sequences from GenBank. The scale bar is in units of nucleotide substitutions per site (s/s) and the tree is mid-pointed rooted. Colors represent different sampling locations. (**C**) Time-scaled maximum clade credibility phylogeny of DENV1-V-MS clade 1 including 82 genomes obtained in this study, plus the 15 genome sequences isolated in Paraguay in 2022. Tips are colored according to sampling location. Values around key nodes represent posterior probability support.; (**D**) Time-scaled maximum clade credibility phylogeny of DENV1-V-MS clade 2 including 88 genomes obtained in this study, plus the 4 genome sequences isolated in Paraguay in 2022. Tips are colored according to sampling location. Values around key nodes represent posterior probability support.

To investigate the phylodynamics of DENV1, we combined our 172 newly sequenced genomes with existing DENV1 genotype V (DENV1-V) genome sequences from GenBank (*n* = 552). Our phylogenetic analysis reveals that the newly identified isolates formed four distinct clades: I, II, III, and IV (Figure 3). These findings point towards a complex transmission dynamic, indicating the involvement of different regions, including the Brazilian Northeast, Southeast, and other South American countries like Paraguay, in the introduction and progression of the DENV1 epidemic in Mato Grosso do Sul state (Figure 3). Within the identified clades, our analysis revealed that the newly sequenced genomes in clades III and IV formed robust monophyletic clusters. This observation suggests community transmission and the persistence of viral strains in the region for approximately six months, highlighting the local circulation and the persistence of these viral strains within the state.

To delve into the evolution of these two clusters further, we utilized smaller datasets specific to each cluster (*n* = 97 for MS cluster I and *n* = 92 for MS cluster II). By examining substitution rate constancy, we found a strong correlation between the sampling time and the root-to-tip divergence in these independent subsets. This correlation allowed us to employ molecular clock models to infer evolutionary parameters. For MS clade I, we obtained a coefficient correlation of 0.8 and an r^2^ value of 0.60, while for MS clade II, the coefficient correlation was 0.6, and the r^2^ value was 0.40.

Through our Maximum Clade Credibility (MCC) tree analysis of MS clade I (Figure 3C), we found that all the novel strains clustered together within a well-supported clade (posterior probability support, PPS = 1.0). This clade also included isolates from Paraguay that were sampled in 2022. The estimated time to the most recent ancestor (tMRCA) for this clade was January 2021, with a 95% highest posterior density (HPD) ranging from July 2019 to August 2021.

Similarly, the MCC reconstructions for MS clade II demonstrated that all the newly sequenced strains clustered into a single well-supported clade (PPS = 1.0). This clade also included isolates from Paraguay sampled in 2022. The estimated tMRCA for this clade was January 2022, with a 95% HPD ranging from December 2021 to February 2022 (Figure 3D).

Both clades, which include strains from Paraguay, a neighboring country to Mato Grosso do Sul, suggest the occurrence of cross-border transmission, emphasizing the region’s interconnectedness and the inherent potential for the exchange of viral strains.

### 3.2. DENV2 Phylodynamics in the State of Mato Grosso do Sul, Midwest Brazil

**Figure 4:**
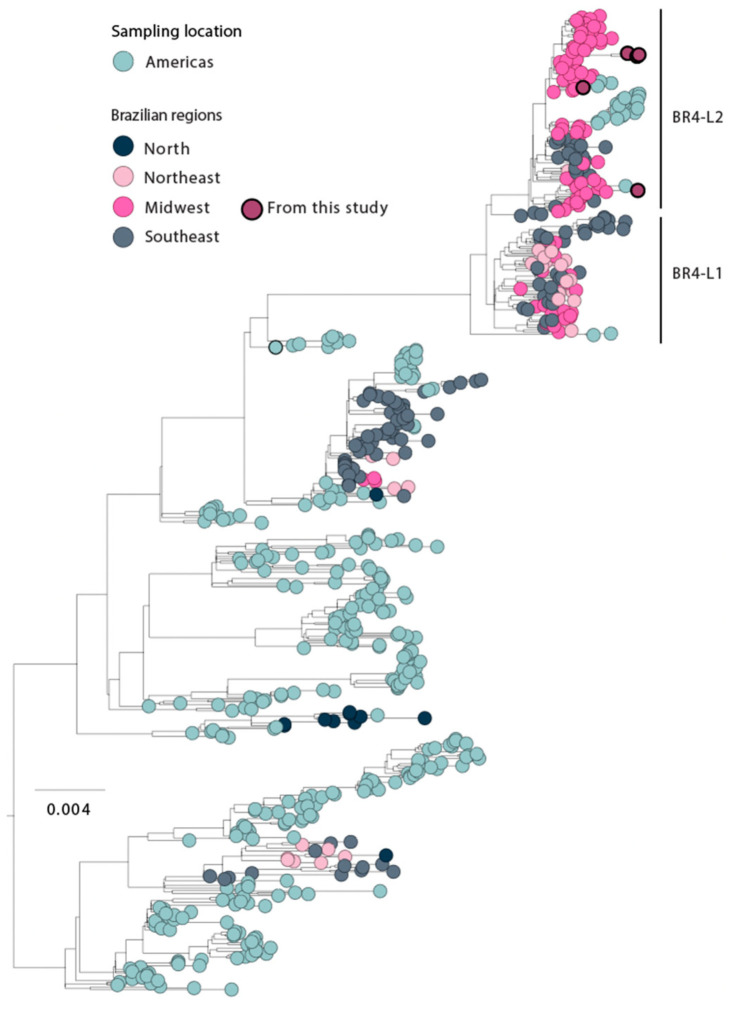
Genomic monitoring of DENV2 genotype III in Mato Grosso do Sul state, Midwest Brazil. Maximum likelihood (ML) phylogenetic analysis of 5 newly near-complete genome sequences from DENV2 generated in this study plus 648 available sequences from GenBank. The scale bar is in units of nucleotide substitutions per site (s/s) and the tree is mid-pointed rooted. Colors represent different sampling locations.

In order to investigate the phylodynamics of DENV2 in Mato Grosso do Sul state, we conducted a phylogenetic analysis involving our newly generated sequences (*n* = 5) and 648 complete genome sequences of DENV2-III obtained from GenBank (Figure 4). 

This analysis revealed that our novel strains belonged to the DENV2 genotype III, BR-4 L2 clade that was initially identified in Brazil towards the end of 2019 and clustered together with genome sequences sampled from different Brazilian regions [1]. It is important to note that the number of genome sequences available from Mato Grosso do Sul belonging to this specific clade is limited. The scarcity of complete genome sequences significantly impacts our estimates and ability to characterize the molecular epidemiology of these viral strains at a regional level. 

## 4. Discussion

Brazil continues to face a significant burden from dengue fever, marked by recurrent outbreaks with varying severity and geographic distribution. The country’s warm climate, urbanization, and dense population create an ideal environment for the spread of this endemic virus. Despite the significant impact of the disease, there remains much to be discovered, particularly in the Midwestern region of the country, regarding the genetic diversity and evolution of circulating DENV strains. Understanding these factors is crucial for effectively addressing the challenges posed by dengue and implementing targeted control and prevention measures.

In order to fill this knowledge gap, we conducted a study in which we generated 177 whole genome sequences from the 2021–2022 DENV epidemic to gain a deeper understanding of the overall DENV landscape in the state of Mato Grosso do Sul. This was made possible through an integrated strategy that included an on-site training program, genetic surveillance, and epidemiological analysis.

After a decline in the number of cases, analysis of the epidemiological data revealed multiple epidemic peaks, each characterized by the circulation of different DENV serotypes. These patterns suggest a gradual development of herd immunity over time. This dynamic interplay accentuates the necessity for sustained surveillance and monitoring of dengue virus serotypes, underscoring their centrality in public health interventions and preventive strategies. Multiple pivotal reasons underscore the significance of continuous surveillance and monitoring initiatives. Foremost among these is the capacity to discern shifts in predominant serotypes circulating within specific populations. Variability in virulence, transmissibility, and potential to induce severe illness across different DENV serotypes necessitates their identification for effective resource allocation and outbreak prediction. Furthermore, genomic analysis of circulating dengue strains might reveal information about the introduction of novel mutations or viral variants. Some of these genetic changes may help the virus avoid detection by immune systems or increase host infectivity. Early detection of such changes allows public health organizations to shift their strategies and develop targeted interventions, such as adapting vaccine formulations to account for new variants. Moreover, incorporating genetic analysis into surveillance practices also empowers international and cross-border collaboration. Given that dengue knows no boundaries and can be easily spread across regions through travel and trade, understanding the genetic relationships between strains in different areas is crucial for coordinated prevention and control efforts. Sharing this data allows neighboring countries to collectively anticipate and manage potential outbreaks, minimizing the virus’s impact.

Our newly generated genome sequences allowed us to identify the co-circulation of different DENV serotypes (DENV1-2), which suggests that distinct viral strains were introduced to the region multiple times. The novel DENV1 strains were classified as genotype V and formed four distinct clades (I–IV), with clades III and IV formed robust monophyletic clusters. These findings suggested community transmission and the persistence of those viral strains for around six months in the state. Notably, we identified a connection between Mato Grosso do Sul and its neighboring country, Paraguay, underscoring the pivotal role of cross-border transmission in facilitating the dissemination of both emerging and re-emerging viral strains. The movement of DENV strains across borders could potentially be aided by factors such as the mobility of infected individuals, trade activities, and tourism. To effectively implement efficient dengue control strategies and curtail the onward spread of the virus, collaborative efforts among the surrounding nations emerge as imperative.

Turning to our findings on DENV2 strains, our analysis detected the presence of genotype III, and we found that our novel strains were closely related to the recently described BR4-L2 lineage [1]. However, due to the limited availability of whole genome sequences for this viral strain, we faced challenges in reconstructing the transmission path of this lineage within the state.

Despite our study highlights the outcomes of phylogenetic and phylodynamic analyses on newly DENV genome sequences from Mato Grosso do Sul, it is crucial also to recognize the temporal gaps and uneven distribution of DENV datasets. These gaps introduce potential biases into our results. The shortage of comprehensive DENV genome sequences across South America significantly impacts our estimations and ability to characterize the molecular epidemiology on a regional scale. Nonetheless, we anticipate that ongoing sequencing initiatives will progressively mitigate this disparity, enhancing the accuracy of our insights. A coordinated approach and ongoing genetic surveillance are required for effective dengue control and preventative management. Recognizing the worldwide importance of this health issue, these proactive efforts resonate throughout various fields, including public health initiatives, vaccine development, and regional collaboration.

## Data Availability

Newly generated DENV sequences have been deposited in GenBank under accession numbers OR258403–OR258579. All inputs and codes used in this study were made available in supplementary files and on the project GitHub repository: https://github.com/genomicsurveillance/dengue_serotype_1V_and_2III_mato_grosso_do_sul.

## Supplementary Materials

The following supporting information can be downloaded at: https://www.mdpi.com/article/10.3390/v15091790/s1, Table S1: Epidemiological data the 177 DENV1-2 samples sequenced as part of this study.
